# A Novel Vitamin E TPGS-Based Formulation Enhances Chlorhexidine Bioavailability in Corneal Layers

**DOI:** 10.3390/pharmaceutics12070642

**Published:** 2020-07-08

**Authors:** Ciro Caruso, Amalia Porta, Alessandra Tosco, Daniela Eletto, Luigi Pacente, Silvia Bartollino, Ciro Costagliola

**Affiliations:** 1 Corneal Transplant Center, Pellegrini Hospital, 80134 Naples, Italy; cirocarusoeye@gmail.com (C.C.); pacente@oculum.it (L.P.); 2 Department of Pharmacy, University of Salerno, 84084 Fisciano, Italy; aporta@unisa.it (A.P.); tosco@unisa.it (A.T.); 3 Department of Medicine and Health Science, “V. Tiberio” University of Molise, 86100 Campobasso, Italy; ciro.costagliola@unimol.it

**Keywords:** chlorhexidine, vitamin E TPGS, keratitis, ocular infections, drug delivery systems

## Abstract

Keratitis is a severe condition characterized by inflammation of the cornea following a local trauma. The most common ocular disease is the bacterial one, which requires an antibiotic treatment. The major limitation of this therapy is the resistance of the antibiotic. For this reason, alternative procedures have been developed and consist of antimicrobial molecules. One of the most used is the chlorhexidine gluconate, which has shown activity versus Gram-positive and Gram-negative bacteria and fungi. In addition to its efficiency, chlorhexidine shows low toxicity levels for mammalian cells and is a low-cost molecule. Despite its multiple benefits, chlorhexidine, if used at concentrations higher than 0.02% (*w/w*), can cause local eye irritation. Additionally, its poor penetrability through the cornea makes necessary frequent instillation of eye drops for a prolonged time. Due to these limitations, alternative drug delivery strategies are required. Here, we report a novel formulation based on the combination of d-alpha-tocopherol polyethylene glycol 1000 succinate with chlorhexidine, which results in higher accumulation of the drug in human corneas measured by liquid chromatography and strong antimicrobial activity. Moreover, this formulation does not cause any toxic effect on human cells and is well tolerated by rabbit eyes. Therefore this novel formulation represents a good candidate for the treatment of keratitis that overcomes the risk of antibiotic resistance.

## 1. Introduction

A healthy cornea is rather resistant to infections; in fact, the corneal epithelium poses a formidable mechanical barrier to most pathogens. Systemic and local factors may enhance corneal susceptibility to infection leading to inflamed conditions named keratitis [1]. Trauma affecting corneal integrity is responsible for the introduction and inoculation of various pathogens into the corneal layers. Wearing, removing, or inserting contact lens represent one of the major risk factors for initiating corneal infection [2,3].

The most common type of ocular infection is the bacterial one and it might cause pain, light sensitivity and ultimately blindness [1]. The current protocol for treating corneal infection is based on an antibiogram and antimycotic analysis before initiating antibiotic therapy [4]. In several studies it has been shown that the microbial spectrum has been changing in recent years, showing a marked increase in *Fusarium* keratitis and filamentous fungi even in countries with a temperate climate [5,6]. The antibiotic-antimicogram analysis then allow to evaluate the in vitro sensitivity of the pathogen isolated to the various agents tested. The strong positive correlation between a high minimum inhibitory concentration (MIC) value and the risk of perforation is also demonstrated [7]. When the infectious forms are poorly sensitive to medical therapy, or in the presence of descemetocele with the risk of imminent corneal perforation, the surgical treatment becomes necessary [8]. The goal of a surgical treatment is to eliminate the infecting elements, and reduce the ongoing immunological reaction as well as the removal of necrotic tissues. Moreover, tissue sampling is necessary for direct microbiological examination and culture [8]. For this purpose, in infectious keratitis corneal scraping, with a surgical blade or platinum spatula, for the collection of the corneal sample, is recommended [9]. Equally recommended is to perform a swab for the collection of the tear film in conjunctival infections [5].

Surgical treatment for small and superficial ulcers involves debridement of infected tissues, superficial keratectomy and tarsorrhaphy. In the presence of severe injuries at the risk of major complications, conjunctival overlay, or perforating keratoplasty are needed, especially when the responsible agents do not answer to medical therapy [10].

Another recently proposed surgical procedure is the corneal cross-linking (CXL). Due to the photochemical activation of riboflavin, CXL allows the formation of collagen bridges, thus strengthening the stromal lamellae [11,12]. The effectiveness of this approach is due to a direct anti-microbial action and, indirectly, to a strengthening of corneal tissues against enzymatic degradation. However, in vitro studies failed to show the ability of CXL alone in the inactivation of bacteria and fungi. Only a study that combined CXL and Amphotericin B demonstrated an in vitro inactivation of fungi [13].

An alternative approach is the use of general antiseptics, which present an extensive broad-spectrum coverage [14]. One of the most commonly used is the chlorhexidine gluconate (CHX), which has shown activity in Gram-positive vs. Gram-negative bacteria and fungi and moreover in *Chlamydia trachomatis* vs. *Achanthamoeba* [15,16]. In some studies, the effectiveness of CHX for topical use has also been validated [17]. Since CHX has strong antimicrobial activity but relatively low toxicity levels for mammalian cells, it is considered the most useful and safest disinfectant. Its propensity to bind to the surface of the tissues offers a long-lasting antimicrobial effect [18,19,20]. CHX, compared to currently used antibiotics, has many advantages. In fact, it provides extensive antibacterial coverage, lower drug resistance, and represents a much more affordable therapy [21]. Despite its multiple benefits, such a therapeutic approach still has some limitations due to its toxicity when used at a concentration above >0.02% (*w/w*) [22,23,24,25,26]. In vitro studies have demonstrated that CHX has a toxic effect on conjunctival and corneal epithelial cells. In clinical studies, at higher concentrations CLX induces corneal injury, edema, desquamation, and delayed healing, whereas at lower concentrations (0.005%), a mild irritation and corneal opacities has been recorded [27]. However, short-term exposure of the culture to the minimal concentrations of CLX resulted in viability rates above 90%. To prevent local eye irritation CHX is commonly used at low concentration and therapy consists of frequent instillation of eye drops for a prolonged time. This kind of intense treatment might cause side effects due to an overdose and, thus, toxicity often is accompanied by reduced compliance of patients.

Another issue for treating keratitis is the poor penetrability of drugs towards the deep corneal stroma, which limits the number of molecules to work with [28]. CHX does not readily penetrate through the cornea into the anterior chamber, clarifying why the treatment of keratitis requires many months of sustained topical drug administration [29].

To address these issues, novel formulations have been developed by using D-a-Tocopherol polyethylene glycol 1000 succinate (TPGS or Vitamin E TPGS)-based strategy [30,31]. This water-soluble molecule is formed by esterification of vitamin E succinate with polyethylene glycol (PEG) 1000. Its amphipathic properties make this nonionic surfactant able to form stable micelles in aqueous vehicles, and help to solubilizing poorly water-soluble molecules [32,33,34]. In addition to increasing bioavailability of many drugs, TPGS consists of a safe molecule and is indeed approved by the Food and Drug Administration (FDA) as adjuvant in drug delivery systems (DDSs).

Vitamin E has no irritant effect on the eye, and its antioxidant properties make it beneficial also for topical application [35,36,37,38,39].

Given the antimicrobial activity of CHX and the beneficial properties of VE-TPGS, we developed for the first time a TPGS-based formulation for extending the CHX penetration throughout the cornea. Our evidence from antimicrobial tests along with in vitro and in vivo toxicity assays strongly show that this novel ophthalmic preparation consists of a very effective drug with high accumulation of CHX in human corneas. 

## 2. Materials and Methods

### 2.1. Materials

A solution based on D-alpha-tocopherol poly (ethylene glycol) 1000 succinate (VE-TPGS; 0.5–0.2%) and CHX digluconate solution (0.02%) from Iromed Group Rome Italy was tested. Distilled and Milli-Q deionized water (DI) was used for all preparations.

### 2.2. Cell Line and Culture Conditions

L929 fibroblasts cells monolayers are highly representative of the target tissue in vivo. L929 cells were cultured in MEM (minimal essential medium) supplemented with fetal bovine serum (10%) and glutamine (4 mM) and incubated at standard culture conditions (37 °C, 5% CO_2_).

### 2.3. Ex Vivo Determination of Corneal Drug Penetration in Franz Diffusion Cells

Corneal accumulation of the solution was evaluated using modified Franz-type diffusion cell (Ø 9 mm, 5 mL receptor volume, SES GmbH—Analysesysteme, Bechenheim, DE). Seven groups of 10 human corneas provided by fast linking (spin-off consortium Fast Linking Naples Italy) were soaked for 20 min in the formulation; six were labeled from B1 to B6. A seventh group, labeled A, used as a control group, was soaked in the same solution as above without VE-TPGS. Corneas were treated as reported in [40]. Different aqueous iso-osmolar solutions, buffered at pH 7.2, were used as donor compartment. They all contained 0.02% *w/w* of CHX and different concentrations of VE-TPGS, as shown in Table 1. Solution A contained only CHX and was used as control. All solutions from B1 to B6 were applied on epithelized corneas in order to evaluate the accumulation of CHX as reported in [40].

### 2.4. HPLC Analysis

The equipment and the analytical settings were the same used in [40,41]. Results are expressed as mean ± SEM. Data obtained were analyzed by Student’s *t* test for statistical significance. A *p* value ≤0.05 was considered significant in this study.

### 2.5. In Vitro Toxicity by 3-(4,5-Dimethylthiazol-2-yl)-2,5-diphenyltetrazolium Bromide (MTT) Assay

VE TPGS CHX formulation was tested for its toxicity on L929 cells by an 3-(4,5-dimethylthiazol-2-yl)-2,5-diphenyltetrazolium bromide (MTT) assay [42]. Briefly, the VE-TPGS CHX formulation containing respectively 0.5% and 0.02% was incubated overnight in a culture medium. A L929 cell suspension was prepared at a concentration of 4 × 10^4^ cell/mL and seeded onto 96-well culture plates (100 μL/each well). Cells were incubated in the medium containing the formulation or in the subsequent dilutions (1:2) in a culture medium for 24 h. Sodium dodecyl sulfate (SDS) was served as a positive control at concentrations between 0.00313 and 0.4 mg/mL, whereas untreated cells in a culture medium were used as a negative control. After a 24 h-period contact, the culture medium was carefully removed from each well and the cells were treated with MTT (1 mg/mL) and incubated for 3 h at standard culture conditions. After this period, the solution of MTT was washed out and the formazan crystal dissolved by 100 μL of isopropanol. The absorbance (optical density, OD) was determined spectrophotometrically at 540 nm wavelength.

### 2.6. In Vivo Ocular Tolerance in Rabbits

A total of twelve (12) female New Zealand white specific pathogen free (SPF) rabbits, approximately 11 weeks old, were supplied from Charles River Italia S.p.A., Calco (Lecco), Italy and all the experiments were carried out by the Research Toxicology Center SPA in Pomezia (Italy) (RTC STUDY NO. A3656). The animals were assigned to two groups of 5 animals, a control group receiving only the placebo and the second one receiving the eye drop. A formulation containing respectively 0.5% and 0.02% of VE-TPGS CHX was instilled in the right eye, twice daily at a 6-h interval. A volume of 60 µL/administration of the test item or control item was instilled into the lower conjunctival sac of the right eye, ensuring that the globe or conjunctivae were not damaged. The eye was then held closed for a few seconds to prevent loss of the formulation and to aid distribution over the surfaces of the eye and conjunctival membranes. The left eyes served as a control. Animals were treated 2 times a day for 7 consecutive days. Animals were dosed up until the day before necropsy. Both eyes of all animals were examined once pre-dose and twice daily thereafter, prior to each administration. Any observed irritation was allocated a numerical value according to the table below based on the methods described by Draize et al. [43]. The study has been conformed to the Association for Research in Vision and Ophthalmology (ARVO) Statement for use of animals in ophthalmic and vision research, and in accordance with the guidelines of the European Economic Community for animal care and welfare (EEC Law No. 86/609). The study received approval from the local Ethics Committee No 21/2019 (22 October 2019).

### 2.7. Microorganisms

The antimicrobial activity of the VE-TPGS CHX formulation was evaluated against *Staphylococcus aureus* (ATCC 6538), *S. epidermidis*, *Pseudomonas aeruginosa* (ATCC 9027), *Escherichia coli* (ATCC 8739), *Candida albicans* (ATCC 10231), and *Aspergillus niger* (ATCC 16404).

### 2.8. Determination of Minimal Inhibitory Concentrations (MICs), Minimum Bactericidal Concentrations (MBCs), and Minimum Fungicidal Concentration (MFC)

To determine the in vitro minimal inhibitory concentrations (MIC) of VE-TPGS CHX formulation, microbroth dilution assays were performed in line with the Clinical and Laboratory Standards Institute (CLSI) guidelines [44]. In details, from overnight cultures in Mueller–Hinton Agar (Becton Dickinson and Company, Franklin Lakes, NJ, USA) bacteria were first resuspended at a concentration of ≈ (approximately equal to) 1.5 × 10^8^ CFU/mL in sterile saline and then further diluted at a concentration of ≈1.5 × 10^5^ CFU/mL in cation adjusted Mueller–Hinton Broth (CAMHB, Thermo Scientific, Waltham, MA, USA) containing several dilutions of VE-TPGS CHX formulation. Plates were incubated in air for 24 h at 37 °C. 

Similarly, the fungistatic and fungicidal activity of VE-TPGS CHX formulation was tested against *Candida albicans* by the micro-broth dilution method in 96-well plates according to the guidelines reported in the CLSI document M27-A3 [45]. In details, colonies from Sabouraud’s dextrose agar plate were grown in pre-warmed RPMI 1640 medium (with l-glutamine, 2% glucose, without bicarbonate, and with phenol red as a pH indicator) buffered to pH 7.0 with 0.165 M morpholine propane sulfonic acid (MOPS). Several dilutions of VE-TPGS CHX formulation were added to each well containing 2 × 10^3^ yeast/mL. The plate was incubated at 37 °C for 24 or 48 h.

The MIC was then determined visually as the lowest concentration showing no growth. The minimum bactericidal concentration (MBC) and the minimum fungicidal concentration (MFC) were determined by plating 20 μL of each well on agar plates incubated at 37° for 24 h or 48 h. The MBC and the MFC were identified as the lowest concentration plated to show no microbial growth. Each assay was performed in triplicate. The MBC/MIC ratios were calculated in order to determine a bactericidal/fungicidal (MBC/MIC ≤ 4 or MFC/MIC ≤ 4) or bacteriostatic/fungistatic (MBC/MIC > 4 or MFC/MIC > 4) effect of this formulation.

### 2.9. Biofilm Inhibition

To determine the ability of VE-TPGS CHX formulation to inhibit the bacteria biofilm formation, we incubated bacteria with several dilutions of the formulation. After 24 h, loosely adherent cells were removed by washing twice with Phosphate Buffered Saline (PBS) and the biofilms were stained for 15 min with 200 μL of crystal violet (CV, 0.3% *w*/*v*). Biofilms were rinsed twice with water and the dissolved with 200 μL of 33% acetic acid. The amount of biomass was quantified by measuring the absorbance of the CV solution in a microplate spectrophotometer set at 600 nm. Untreated cells were used as a control. Each assay was performed in triplicate on separate days.

Similarly, yeast cells were incubated in a yeast nitrogen base with 100 mM glucose at 37 °C for 90 min. Loosely adherent cells of *C. albicans* were removed by washing with PBS, while adherent cells were incubated with different dilutions of the formulation. After 48 h, the biofilm mass was washed with PBS, dried, stained with CV for 15 min, and quantified by recovering biomass with 200 μL/well of 33% acetic acid.

### 2.10. Challenge Test

The microbial barrier properties of the formulation were evaluated by an in vitro challenge test described by the European Pharmacopeia to estimate potential contaminations during its use. The formulation was tested against the following bacteria and fungi: *Staphylococcus aureus* (ATCC 6538)*, Pseudomonas aeruginosa* (ATCC 9027), *Candida albicans* (ATCC 10231) and *Aspergillus niger* (ATCC 16404). In details, according to the standard methodology, the formulation was split into 10 mL aliquots, which were inoculated with 10^6^ and 10^5^ cells/mL bacteria and fungi respectively, and incubated at 20–25 °C. At different time points, 2, 7, 14, and 28 days, one mL aliquots were serially diluted in tryptone-azolectin-Tween broth and plated in duplicate on tryptic-soy agar (for bacteria) or Sabouraud dextrose agar (for fungi). Plates were incubated at 30–35 °C for ≥3 days for bacteria and 20–25 °C for ≥5 days for fungi. Raw data counts were converted to log_10_ values.

## 3. Results

### 3.1. VE-TPGS Increases Corneal Accumulation of CHX

To evaluate whether this novel formulation increases the delivery of CHX throughout the cornea, different combinations of CHX and VE-TPG were applied on human corneas and analyzed by a modified Franz-type diffusion cell system. In particular, seven groups of 10 human corneas were incubated for about 20 min with 0.02% (*w/w*) CHX and an increasing % (*w/w*) of VE-TPGS (from 0 to 1) as shown in Table 1.

Then, corneas were processed according to the procedure reported by Ostacolo et al. and CHX permeability was determined by assaying the recipient chamber over time and quantifying the molecule by HPLC [40]. The accumulation of CHX was expressed as nmol of CHX per mg of corneal tissue as reported in Table 2. Solution A was used as a control.

In all the analyzed conditions, except the one named B1, we measured higher concentration of CHX compared to the control (A). Interestingly, at increasing concentrations of VE-TPGS there was higher accumulation of CHX up to the condition B5 where VE-TPGS was used at 0.5% (*w/w*). No significant differences were found between B5 (0.5% *w/w* VE-TPGS) and B6 (1% *w/w* VE-TPGS) conditions.

VE-TPGS’ concentration should not exceed the critical micelle concentration (CMC), as over certain threshold biocidal activity of ophthalmic preservatives drops down significantly [46]. Furthermore, higher concentration of non-ionic surfactants might lead to potential safety risk for disruption of the precorneal tear film [47]. Due to these reasons, condition B5 was used for the following analysis.

### 3.2. In Vitro Toxicity of VE-TPGS CHX Formulation

Once an improved delivery of CHX through the cornea was established, the VE-TPGS CHX formulation was assayed for its toxicity in vitro. A novel eye drop is usually tested for being able to stimulate in vitro the growth of cells and for being compatible with their metabolism with non-cytotoxic effects, even at relatively high concentrations. The results would represent an index of the high skin compatibility of a medical device and also suggest a potential role of the device itself in accelerating the turn-over of the skin on which the topical products are directly applied [48,49].

**Figure 1:**
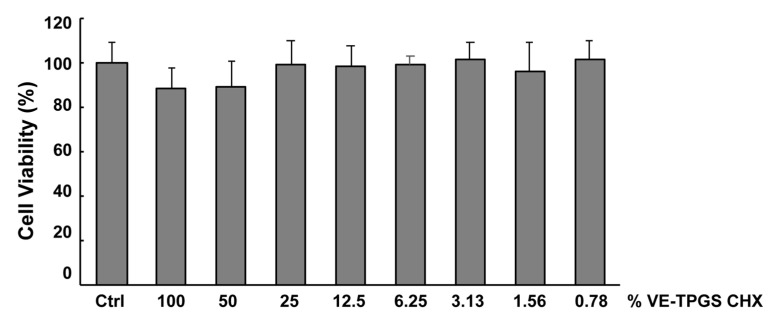
VE-TPGS CHX formulation is not cytotoxic. The formulation of VE-TPGS (0.5% *w/w*) and CHX (0.02% *w/w*) was tested on L929 cells by MTT assay. Cells were incubated for 24 h with the formulation (100%) or its serial dilutions. Each condition was performed in triplicate and the experiment repeated three times. The cell viability of the treated cells is expressed as percentage of the control (Ctrl). The Student’s *t*-test shows no significant difference among all the conditions.

The cytotoxicity assay was performed on L929 fibroblasts cells, which were treated with liquid of maceration and subsequent 1:2 dilutions. Liquid of maceration was obtained after incubating overnight the formulation of VE-TPGS (0.5% *w/w*) and CHX (0.02% *w/w*) in culture medium. After 24 h of incubation, cells were analyzed by an MTT assay. As shown in Figure 1, the viability of the cells post-incubation with the extract (100% liquid of maceration) and the serial dilutions was not statistically affected, suggesting that this formulation does not cause cytotoxic effects or stimulate cell proliferation.

### 3.3. In Vivo Ocular Tolerance Study in Rabbits

The tolerance of VE-TPGS CHX formulation was also tested in vivo by two daily ocular instillations of 60 µL into the right eye of six rabbits for 7 days at the Research Toxicology Center (RTC STUDY NO. A3656). The left eye of each animal remained untreated and served as an intra-animal control. As the control group, another set of six rabbits was treated with 0.9% sodium chloride. Twice per day, in each animal were observed and recorded the following parameters: clinical signs, body weight, ocular observations (twice daily ocular irritation assessment), and post mortem gross macroscopic observations (with particular attention to the eye and adnexa). None of the rabbits receiving VE-TPGS CHX developed signs of systemic effects. Additionally, the examination of both eyes pre- and post-dose of VE-TPGS CHX did not reveal any treatment-related effect or irritation. Indeed, we assigned to each animal a score of zero in a range from 0 to 4 where 0 corresponds to the normal physiology of the eye and 4 to the most signs of irritation according to Draize et al. (data not shown) [43]. No treatment-related changes were observed at post mortem macroscopic observations (data not shown).

Together, the results strongly suggest that this novel ophthalmic solution is well tolerated by the eye of the rabbit, in two daily administrations of 60 µL onto the surfaces of the eye.

### 3.4. Antimicrobial Activity of VE-TPGS Chlorhexidine Gluconate (CHX) Formulation

In order to verify if the antimicrobial activity of CHX combined with VE-TPGS was preserved, MIC and MBC of the drug were evaluated against Gram-positive and Gram-negative bacteria strains (*Staphylococcus aureus*, *Staphylococcus epidermidis*, *Pseudomonas aeruginosa*, and *Escherichia coli*) and fungi (*Candida albicans*). As shown in Table 3, all the bacteria but *P. aeruginosa* and *C. albicans* were significantly inhibited in their viability and among them, the strongest effect was against *S. aureus* with the lowest MIC (9 μL/mL) and MBC (35 μL/mL) values (Table 3). The MBC/MIC ratios showed that the formulation has bactericidal effect on *S. aureus*, *S. epidermidis*, *E. coli*, as well as a fungicidal effect on *C. albicans*, with all MBC/MIC values below 4 (Table 3).

**Figure 2:**
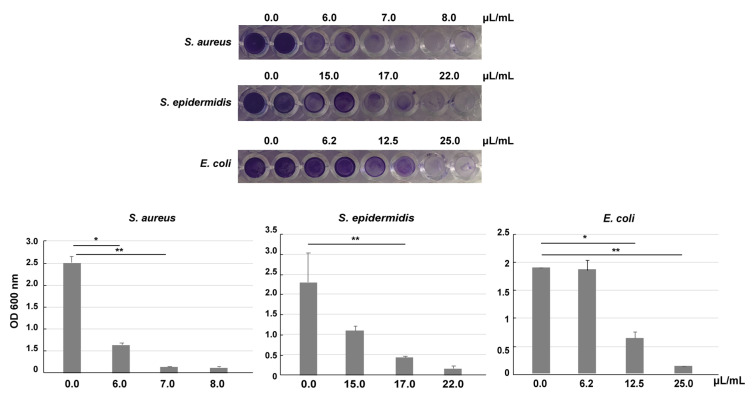
Inhibition of biofilm formation by VE-TPGS CHX formulation. *S. aureus, S. epidermidis* and *E. coli* were grown for 24 h in presence of different dilutions of the formulation. Biofilms were stained by crystal violet, dissolved and measured at 600 nm. The results are expressed as mean ± SEM. * *p* ≤ 0.05, ** *p* ≤ 0.01, the data were analyzed by student’s *t*-test.

Moreover, the effectiveness of the preparation was tested by the ability to inhibit biofilm formation (Table 3 and Figure 2). The biofilm formation assay mirrored the results of MIC and MBC, with a major and predominant effect on Gram-positive bacteria (ranging from 6/7 to 17 μL/mL), whereas among the Gram-negative bacteria only *E. coli* (>12.5/25 μL/mL) was affected by the treatment. 

**Figure 3:**
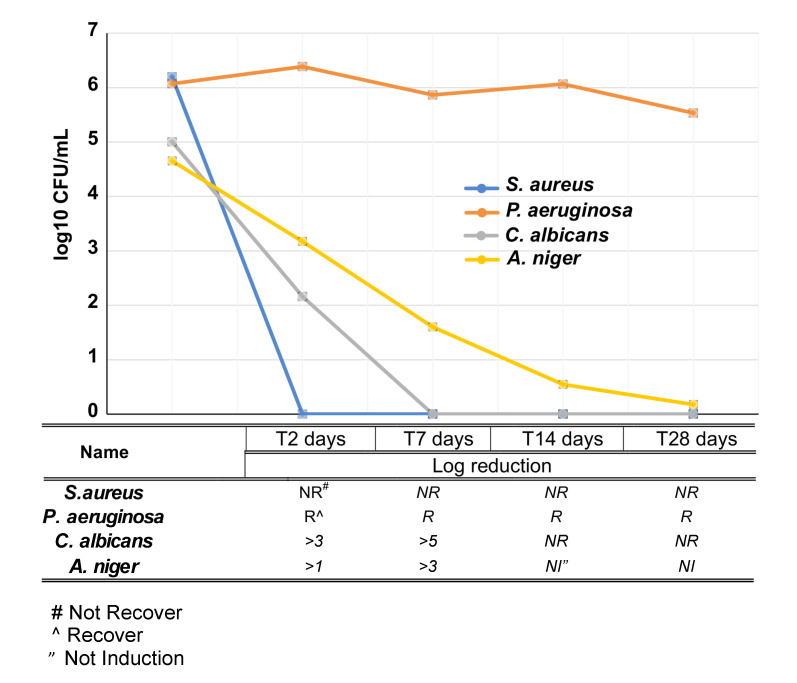
Challenge test over VE-TPGS CHX formulation. The VE-TPGS CHX formulation was challenged by *S. aureus, P. aeruginosa, C. albicans and A. niger*. At the indicated time points, inoculated solutions were collected and plated. After 2/3 days of incubation, colonies were counted and counts converted to log_10_ values.

The ability of the formulation to prevent microbial growth was tested by the challenge test, in which the formulation was directly inoculated with *S. aureus*, *P. aeruginosa*, *C. albicans*, and *A. niger* and their growth monitored over time by counting their colonies on agar plates. As shown in Figure 3, among the bacteria only *S. aureus* was fully inhibited the whole time, while the fungi, *C. albicans* and *A. niger*, were reduced respectively at 2 days by 3- and 1-fold and at 7 days by 5- and 3-fold. At 14 days *C. albicans* did not recover anymore, while *A. niger* maintained the same growth rate observed at 7 days. All together, these evidences suggest that VE-TPGS CHX formulation meets the typical needs of antimicrobial eye drops.

## 4. Discussion

CHX is a broadly spectrum antiseptic chemical disinfectant, active against both Gram-positive/Gram-negative bacteria and fungi. Its bactericidal action is due to a strong increase of the permeability of the bacterial cell membrane, resulting in a modification of its protein structure, with a consequent precipitation of several cytoplasmic macromolecules and finally cell death by lysis [50,51].

CHX is the selective molecule for keratitis because it is efficient and well tolerated under certain levels (<0.02%) and it does not cause antibiotic-related resistance. Despite its many advantages, its use is limited by poor penetration through the cornea stroma. It is generally recognized that the barrier function to the epithelium resides on the external surface and that the permeation enhancers act by changing the permeability of the surface cells. In the group of permeation promoters, nonionic surfactants are really well known for their effectiveness, ease of use in aqueous media, and general safety, although their molecular mechanism remains mainly unknown. Among these surfactants, d-alpha-tocopherol poly (ethylene glycol) 1000 succinate (TPGS) is a well-known adjuvant, approved by FDA and has also been described for its protective role on the biological membrane against free radicals [31]. TPGS is a water-soluble derivative of natural vitamin E, formed by esterification of vitamin E succinate with polyethylene glycol (PEG) 1000. A variety of both water-soluble and water-insoluble compounds may be so solubilized, and it has been already used in a wide range of conditions (absorption enhancer, permeation enhancer, stabilizer, emulsifier, and solubilizer) to maximize drug effects reducing the toxic profile. Vitamin E TPGS provides an encapsulation of drugs and through a P-glycoprotein (P-gp) inhibitor activity (P-gps are prominent efflux transporters identified on ocular tissues) improves the ocular absorption of topically administered drugs throughout the cornea [52].

Several clinical evidences support the use of VE-TPGS in ophthalmic preparations for treating eye diseases. Fato et al. demonstrated that VE-TPGS solubilizes Coenzyme Q10, which topically applied, goes across the cornea and can be found in vitreous [53]. VE-TPGS also enhances the corneal penetration of riboflavin-5′-phosphate by modifying the permeability of superficial cells and therefore of the epithelium [40]. Additional advantages from vitamin E include the reduction of degradation rates of various ophthalmic drugs, which is an added benefit of this technology [54].

The addition of vitamin E in the formulation is helpful also for its chemical features. Keratitis is defined as any inflammation of the cornea, regardless of whether it was caused by an infection or an injury. Corneal inflammation is characterized by a cellular and biochemical reaction, which lead to producing arachidonic acid and reactive oxygen species (ROS). Tear fluid contains several antioxidants to protect the ocular surface against certain radicals, such as ascorbic acid, lactoferrin, uric acid, and cysteine. The degree of corneal involvement is due to the balance between ROS and local antioxidant defense system [55]. Vitamin E is the major membrane-associated chain-breaking antioxidant in all tissues and is considered to be the first line of defense against ROS and lipid peroxidation [56]. Its use as an adjuvant in the treatment of keratitis was already known in the early 1940s [57]. The effects of ROS on corneal health consist in tissue damage realized by a reaction with lipid components of the cell membranes, nucleic acids, and sulfur-containing enzymes; topically applied vitamin E is able to reduce these deleterious outcomes [58]. Moreover, in an in vitro study, Satterfield et al. demonstrated that vitamin E inhibits collagen production in corneal keratocytes, indicating a possible further effect of this compound on scar formation, as a regulator of wound healing [59].

The novel combination of VE-TPGS CHX for treating corneal keratitis is helpful. The goal of this formulation is to increase the bioavailability and effectiveness of CHX, and finally to counteract the production of ROS. Our findings demonstrated that VE-TPGS was a good candidate for overcoming irritation due to high concentrations of CHX and promoting a deeper stromal penetration of CHX.

Antimicrobial activity, an in vivo ocular tolerance study in rabbits, in vitro toxicity, and higher corneal accumulation of CHX in VE-TPGS CHX formulation were studied in this paper.

The quantitation of corneal penetration of VE-TPGS CHX in Franz Diffusion Cells followed by an HPLC analysis reported a 10-fold increase in CHX accumulation when combined with VE-TPGS compared to the drug alone. The results confirmed the promising effect of VE-TPGS as an adjuvant for bettering CHX delivery.

The novel formulation was next tested for its toxicity and tolerance in vitro, on L929 fibroblast cells, and in vivo, on the rabbit eye. Cell viability did not turn to be affected significantly by the formulation and rabbit eye treated twice daily for 7 days did not develop any signs of irritation or systemic issues. The novel formulation therefore observes all the safety assessment and toxicological profiling of the drugs.

The most important requirement to meet was the antimicrobial activity. We assessed the effect of this ophthalmic solution in vitro against Gram-positive and Gram-negative strains and fungi. As is known, ocular infections are commonly caused by mostly *Staphylococcus aureus, S. epidermidis*, and *P. aeruginosa*. In particular, *P. aeruginosa* is the worst pathogen for contact lens-related microbial keratitis, while *S. epidermidis* is the first etiologic agent of postoperative endophthalmitis [60,61]. In non-contact-lens-related disease, bacterial keratitis is mostly caused by Gram-positive bacterium [62]. In all these cases a prompt and effective empiric antimicrobial therapy is required.

Here, our results show that VE-TPGS CHX has strong antimicrobial activity particularly in Gram-positive bacteria vs. fungi. In particular, *S. aureus* turned to be the most affected bacterium as proven by the lowest values of MIC and MBC, and the most inhibited in forming biofilm. The formulation was also pretty resistant to contamination when challenged directly with bacteria and fungi, corroborating the preservation of anti-microbial activity. Our evidences are in agreement with previous studies, showing that Gram-positive bacteria are more susceptible than Gram-negative, when exposed to the same concentration of the tested drug [63,64]. *P. aeruginosa* is intrinsically resistant, as the outer membrane restricts the access of CHX to its target [65]. This resistance could be due to a progressive adaptation of the cell membrane induced by progressive mutations resulting in a different composition and changes in intra-cellular biochemical processes [66]. What caused the development of CHX resistance in *P. aeruginosa* is likely due to exposure to subliminal concentrations [67].

## 5. Conclusions

The threat of microbial evolution of antibiotic resistance is a serious concern for the global community. For this reason, attempts are being made to develop antimicrobial strategies that discourage the evolution of this resistance. A well-established alternative to antibiotics is represented by an antimicrobial molecule, named chlorhexidine gluconate (CHX). CHX is the selective drug for keratitis that provides an efficient and affordable therapy. Despite its benefits, CHX’s use is limited because its penetrability through the cornea is poor and at high concentrations causes irritation. For these reasons, we combined CHX with d-alpha-tocopherol poly (ethylene glycol) 1000 succinate (TPGS) in order to develop a novel therapeutic strategy that would overcome the aforementioned CHX’s limitations. As we reported, this novel formulation provides higher accumulation of the drug through the cornea without a toxic effect and preserves all the antimicrobial properties.

We finally present VE-TPGS CHX as a good candidate for keratitis treatment without triggering the risk of antibiotic resistance for eye infections.

## Figures and Tables

**Table 1:** List of donor corneas incubated with different combinations of CHX and VE-TPGS.

Formulation Code	CHX%	VE TPGS%
A	0.02	-
B1	0.02	0.010
B2	0.02	0.050
B3	0.02	0.100
B4	0.02	0.250
B5	0.02	0.500
B6	0.02	1.000

**Table 2:** Quantitation of CHX post cornea accumulation by HPLC analysis.

Formulation Code	CHX Accumulation (nmol/mg)
A	0.032 ± 0.017
B1	0.056 ± 0.033
B2	0.104 ± 0.011
B3	0.213 ± 0.027
B4	0.297 ± 0.031
B5	0.345 ± 0.041
B6	0.376 ± 0.022

Mean CHX accumulation ± SEM at different concentrations of VE-TPGS.

**Table 3:** MIC, MBC and MFC of VE-TPGS CHX formulation ^#^.

Bacteria	MIC	MBC	MBC/MIC	Biofilm Inhibition
*S. aureus*	9.0	35.0	3.9	7.5
*S. epidermis*	27.5	75.0	2.7	17.5
*E. coli*	30.0	40.0	1.3	25.0
*P. aeruginosa*	>100	>100	-	>100
**Fungus**	**MIC**	**MFC**	**MFC/MIC**	
*C. albicans*	50.0	75.0	1.5	

^#^ The VE-TPGS and CHX used in this assay were respectively 0.5% (*w/w*) and 0.02% (*w/w*). MIC, MBC, MFC and Biofilm Formation were all expressed as µL/mL.
